# The Anti-Tumor Effects and Molecular Mechanisms of Suberoylanilide Hydroxamic Acid (SAHA) on the Aggressive Phenotypes of Ovarian Carcinoma Cells

**DOI:** 10.1371/journal.pone.0079781

**Published:** 2013-11-13

**Authors:** Shuo Chen, Yang Zhao, Wen-feng Gou, Shuang Zhao, Yasuo Takano, Hua-chuan Zheng

**Affiliations:** 1 Department of Gynecology, the First Affiliated Hospital of China Medical University, Shenyang, China; 2 Department of Biochemistry and Molecular Biology, College of Basic Medicine, China Medical University, Shenyang, China; 3 Clinical Cancer Institute, Kanagawa Cancer Center, Yokohama, Japan; Queen's University Belfast, United Kingdom

## Abstract

Histone deacetylase inhibitors (HDACi), such as suberoylanilide hydroxamic acid (SAHA), have been shown to act selectively on gene expression, and are potent inducers of growth arrest, differentiation and apoptosis in various types of cancers *in vitro* and *in vivo*. This study aimed to elucidate the anti-tumor effects and molecular mechanisms of SAHA on the aggressive phenotypes of ovarian carcinoma. Two pairs of cell lines (SKOV3 and SKOV3/DDP; HO8910 and HO8910-PM) were exposed to SAHA treatment, and the effects on acetyl-Histone H3 and H4 expression levels were analyzed and compared against the aggressive behaviors of ovarian carcinoma. Our results showed that SAHA suppressed proliferation in both a concentration- and time-dependent manner in all four cell lines; induced S/G_2_ arrest in SKOV3 and SKOV3/DDP cells; and conversely, induced G_1_ arrest in HO8910 and HO8910-PM cells. SAHA treatment induced apoptosis and reduced migration, invasion and lamellipodia formation in the ovarian carcinoma cells; furthermore, SAHA decreased expression of *Cyclin B1* and *CDC2P34* mRNA, and downregulated CDC2P34, Erk1/2, CyclinB1 and MMP-9 proteins. In contrast, SAHA increased expression of Caspase-3, *p21* and *p53* mRNA, and upregulated acetyl-Histones H3 and H4, Caspase-8, and p53 proteins. Basal acetylation of histone H3 and H4 was higher in ovarian carcinoma compared to normal ovarian tissues and benign ovarian tumors, and in borderline tumor than in normal ovarian tissues, and was positively correlated with differentiation and expression of the proliferative marker, Ki-67 (*P* < 0.05). We suggest that SAHA may suppress growth, migration and invasion in ovarian carcinoma cells, including cisplatin-resistant or highly-invasive ovarian cells, by promoting histone acetylation and modulating their phenotype-related molecules. As such, aberrant acetylation of histone H3 and H4 may play an important role in the carcinogenesis and differentiation of ovarian carcinoma.

## Introduction

Histone deacetylases (HDAC) work in concert with histone acetyltransferases (HAT) to regulate gene expression as transcriptional corepressors and activators, respectively. They modify chromatin structure by altering nucleosome conformation, and the stability of several large transcription factor complexes. HDAC inhibitors (HDACi), such as trichostatin A (TSA) have been shown to act selectively on gene expression and are potent inducers of growth arrest, differentiation and apoptosis in transformed cells *in vitro* and *in vivo*.

Suberoylanilide hydroxamic acid (SAHA) is a synthetic hydroxamic acid that has been found to arrest cell growth in a variety of transformed cells *in vitro* at concentrations between 2 and 5 mM [1]. Although SAHA inhibits class I and II zinc-binding HDACs, it does not inhibit class III HDACs. It has been suggested that the mechanism involves SAHA binding to the HDAC-like protein via its hydroxamic acid group that doubly coordinates to a zinc atom at the bottom of the catalytic cavity [2,3]; thereby altering gene expression and protein functions associated with regulation of cell proliferation and cell death pathways. There is considerable evidence suggesting that SAHA induces acetylation of histones and non-histone proteins within transcription factors that regulate gene expression of molecules related to aggressive phenotypes, such as those involved in proliferation, apoptosis, cell motility and angiogenesis[1–3].

Ovarian cancer is one of the leading cause of cancer-related deaths in women worldwide, and is a serious threat to women's health. Ovarian cancers are believed to originate in the ovarian epithelium due to risk factors include genetic mutations and a family history of ovarian cancer. Ovarian cancer is disproportionately fatal due to the lack of chemotherapeutic agents that target ovarian cancer cells. Consequently, most ovarian cancers are metastatic or recurrent. Currently, the five-year survival rate is 47% [4,5]. Here, we observed the effects of SAHA on the phenotypes and relevant molecules of ovarian carcinoma cells, especially drug-resistant or highly-invasive cells. To clarify the clinicopathological significance of aberrant acetylation of histones H3 and H4 in response to SAHA (vorinostat) was determined in ovarian precancerous and cancerous samples by Western blot and immunohistochemisty and compared with clinicopathological parameters of ovarian carcinomas. 

## Materials and Methods

### Cell culture

 Ovarian carcinoma cell lines, HO8910 (serous cystadenocarcinoma), HO8910-PM (invasive adenocarcinoma) and SKOV3 (serous papillary cystadenocarcinoma) were purchased from the ATCC (Manassas, VA, USA); SKOV3/DDP (cisplatin-resistant SKOV3) was purchased from Tumor Cell Bank of the Chinese Academy of Medical Science (Peking, China). The cells were maintained in RPMI-1640 (HO8910; HO8910-PM; SKOV3/DDP) or McCoy's 5A (SKOV3) media, supplemented with 10% fetal bovine serum (FBS), 100 U/mL penicillin and 100 μg/mL streptomycin (SKOV3/DDP was also supplemented with 20ng/ml DDP) in a humidified atmosphere of 5% CO_2_ at 37°C. All cells were harvested by centrifugation, rinsed with phosphate buffered saline (PBS), and subjected to total protein and RNA extraction.

### Proliferation assay

Cell counting Kit-8 (CCK-8; Dojindo, Kumamoto, Japan) was employed to determine the number of viable cells. In brief, 2.5 × 10^3^ cells/well were seeded into 96-well plates and allowed to adhere. At specified time points 10 μL of CCK-8 solution was added to each well and the plates were incubated for a further 3 h in an atmosphere of 5% CO_2_ at 37°C. The number of viable cells was counted by measuring the absorbance at 450 nm using a microplate reader. IC_50_ (half maximal inhibitory concentration of a substance) and CDI (coefficient drug interaction), the coefficient of drug interaction (CDI) was used to analyze the synergistically inhibitory effect of drug combinations. CDI is calculated as follows: CDI = AB/ (A*B). According to the absorbance of each group, AB is the ratio of the combination groups to the control group; A or B is the ratio of the single agent groups to the control group. Thus a CDI value less than, equal to or greater than 1 indicates that the drugs are synergistic, additive or antagonistic, respectively. CDI less than 0.7 indicate that the drugs are significantly synergistic [30].

### Cell cycle analysis

After incubation for 48 h at 37°C in an atmosphere of 5% CO_2,_ cells were detached by trypsinization, collected, washed twice with PBS and fixed in 10 mL of ice-cold ethanol(70%) for at least 2 h. The cells were washed twice with PBS again and incubated with 500μl RNase (0.25 mg/mL) at 37°C for 30 min. The cells were pelleted, resuspended in propidium iodide (PI) at a concentration of 50µg/mL, and incubated in the dark at 4°C for 30 min. Cell cycle analysis was performed by analysis of PI staining by flow cytometry.

### Apoptosis assay

Flow cytometry was performed following staining with PI and FITC-labeled annexin V (KeyGEN Biotech, Nanjing, China) following the manufacturer's instructions to detect phosphatidylserine externalization as an endpoint indicator of early apoptosis in the cells. In brief, after incubation for 48 h at 37°C in an atmosphere of 5% CO_2,_ cells were washed twice with ice-cold PBS, resuspended in 1 × Binding Buffer at a concentration of 1 × 10^6^ cells/mL, and then incubated with 200 μL 1 × Binding Buffer and 10 μL FITC-Annexin V. Samples were gently vortexed and incubated for 15 min at 25°C in the dark, then 300 μL 1 × Binding Buffer and 5 μL PI was added to each tube, Samples were gently vortexed and incubated less than 1h at 25°C in the dark. Flow cytometry was performed within 1 h of incubation.

### Wound healing assay

 Cells were seeded at a density of 1.0×10^6^ cells/well in 6-well culture plates. After they had grown to confluence, the cell monolayer was scraped with a pipette tip(200ul) into 9 areas to create a scratch, washed by PBS for three times and cultured in the FBS-free medium. Cells were photographed at 0,12,24,48,72 h(n=9) and the scratch area was measured using Image software. The wound healing rate = (Area of original wound - Area of actual wound at different times)/ Area of original wound × 100%. 

### Cell invasion assays

For the invasion assay, 5 × 10^4^ cells were resuspended in serum-free RPMI-1640 and seeded into the top chamber of Matrigel-coated transwell inserts (BD Bioscience, San Jose, CA, USA). The lower compartment of the chamber contained 10% v/v FBS as a chemoattractant. After incubation for 48 h at 37°C in an atmosphere of 5% CO_2_, the cells on the upper surface of the membrane were wiped away, and the cells on the lower surface of the membrane were washed with PBS, fixed in 100% methanol and stained with Crystal violet dye to quantify the extent of invasion.

### Immunofluorescence

Cells were grown on glass coverslips, fixed with PBS containing 4% formaldehyde for 10 min, and permeabilized with 0.2% Triton X-100 in PBS for 10 min at room temperature. After washing with PBS, the cells were incubated overnight at 4°C with Alexa Fluor 594 Phalloidin (Invitrogen, Carlsbad, CA, USA) to enable the lamellipodia to be visualized. Nuclei were stained with 1 μg/mL DAPI (Sigma-Aldrich St. Louis, MO, USA) for 15 min at 37°C. The coverslips were then mounted with SlowFade Gold Antifade Reagent (Invitrogen) and observed under a confocal laser microscope (Olympus, Tokyo, Japan).

### Selection of patient samples

Samples of normal ovarian tissue, benign and borderline tumors, primary carcinoma, and metastatic carcinoma in the omentum were collected from patients undergoing surgical resection between January 2003 and December 2011 at Department of Gynecology, The First Hospital Affiliated to China Medical University, Shenyang, China. The average age of the patients at surgery was 51.6 years (range 20–81 years). The majority of samples were routinely prepared for storage in pathological blocks; the remaining samples were immediately frozen in liquid nitrogen and stored at -80°C until required. None of the patients had undergone chemotherapy, radiotherapy or adjuvant treatment prior to surgery. Patients were followed up by consulting their case documents and by telephone. Informed written consent was obtained from all participants and the study was approved by China Medical University Ethics Committee.

### Pathology and tissue microarray (TMA) analysis

All tissues were fixed in 10% neutral formalin, embedded in paraffin and sliced into 4 μm sections. The sections were stained with hematoxylin-and-eosin (HE) to for histological analysis. The clinicopathological staging value was evaluated for each ovarian carcinoma sample according to the International Federation of Gynecology and Obstetrics (FIGO) staging system, based on the extent of tumor spread. Tumor histology was determined according to the World Health Organization (WHO) classification system.

Representative areas of solid tumors were identified in the HE-stained slices of selected tumor samples and tissue cores, 2 mm in diameter, were punched out from each donor block and transferred to a recipient block using a Tissue Microarrayer (AZUMAYA KIN-1, Tokyo, Japan). Each recipient block had a maximum of 48 cores. Consecutive 4 μm sections were incised from the recipient block and transferred to poly-lysine-coated glass slides. HE staining was performed on each tissue microarray (TMA) for confirmation of tumor tissue type.

### Real-time reverse transcriptase–polymerase chain reaction (real-time RT-PCR)

Total RNA was extracted from the ovarian carcinoma cell lines and ovarian tissue samples using Trizol (Takara, Kyoto, Japan) Real-time RT-PCR was performed from 2 µg of total RNA using AMV reverse transcriptase and random primers (Takara). PCR primers were designed according to the sequences in GenBank and are listed in Table S1 in File S1. Amplification of cDNA was performed using the SYBR Premix Ex Taq II kit (Takara), using *GAPDH* as an internal control, according the supplier’s protocol. In brief, RT-PCR amplification of cDNA for each primer was carried out in a final volume of 20 µL containing 10 µL SYBR Premix Ex Taq (×2), 0.08 µL primers, 0.4 µL ROX Reference Dye and 1 µL template cDNA (50 µg/µL). The protocol parameters were as follows: initial incubation at 95°C for 30 s followed by 40 cycles of denaturation at 95°C for 5 s and annealing at 60°C for 34 s. All the PCR experiments were accompanied with no-template control and GAPDH as internal control. Relative gene expression level (the amount of target, normalized to endogenous control gene) was calculated using the comparative Ct method formula 2^-ΔΔCt^. The sequences of primers for real-time quantitative PCR are in Table S1 in File S1.

### Western blot analysis

 Protein assays were performed by the Bradford method using the Bio-Rad protein assay kit (Bio-Rad, Hercules, CA, USA). Denatured proteins were separated by sodium dodecyl sulfate-polyacrylamide gel electrophoresis (SDS-PAGE) on 12% acrylamide gels, and then transferred to Hybond membranes (Amersham, Amersham, Germany). The membranes were blocked overnight in 5% skimmed milk in Tris-buffered saline with Tween 20 (TBST, 10 mM Tris-HCl, 150 mM NaCl, 0.1% Tween 20). For immunoblotting, the membranes were incubated for 1 h with the primary antibody (Table S2 in File S1), rinsed with TBST, and incubated with anti-rabbit, anti-mouse or anti-goat IgG antibodies conjugated to horseradish peroxidase (HRP; Dako, Carpinteria CA, USA) at a dilution of 1:1000. After applying electrochemiluminescent (ECL)-Plus detection reagents (Santa Cruz Biotechnology, Santa Cruz, CA, USA), the protein bands were visualized using X-ray film (Fujifilm, Tokyo, Japan). The immunoblots were washed with Western blotting (WB) Stripping Buffer (pH 2–3; Nacalai, Tokyo, Japan) and probed using a monoclonal antibody specific for β-actin (1:1000; Santa Cruz Biotechnology). Densitometric quantification of acetyl-histone H3 and H4 protein expression in the ovarian samples was performed using Scion Image software, with β-actin as a control.

### Immunohistochemistry

Consecutive sections of tissue samples were deparaffinized with xylene, rehydrated with alcohol, and subjected to antigen retrieval by heating in target retrieval solution (TRS; Dako) for 15 min in a microwave oven (Oriental Rotor Ltd. Co., Tokyo, Japan). The sections were quenched with 3% hydrogen peroxide for 20 min to block endogenous peroxidase activity. Non-specific binding was prevented by adding 5% Bovine Serum Albumin (BSA) for 5 min. The sections were incubated for 15 min with rabbit anti-acetyl-histone-3 (Lys 9/14; Santa Cruz Biotechnology), anti-acetyl-histone H4 (Lys 8; Santa Cruz Biotechnology) or anti-ki-67 (Dako) antibody; then incubated with anti-rabbit antibodies conjugated to HRP (Dako) for 15 min. All incubations were performed by heating in a microwave oven, as described previously [6]. After each treatment, the slides were washed three times with TBST for 5 min, and the binding sites were visualized with 3,3'-diaminobenzidine (DAB). After counterstaining with Mayer’s hematoxylin, the sections were dehydrated, cleared and mounted. Negative controls were prepared by omitting the primary antibody.

Three independent observers (CS, YZ and ZHC) randomly selected and counted 100 cells from five representative fields from each section. Any discrepancies were checked by three observers until a consensus was reached. Positive expression was graded as follows: 0 = negative; 1 = 1%-50%; 2 = 51%-74%; 3 ≥ 75%. The staining intensity was graded as follows: 1 = weak; 2 = intermediate; 3 = strong. The two grades were multiplied to obtain a final score: - = 0; + = 1-2; ++ = 3-4; +++ = 6-9).

### Statistical analysis

Statistical evaluation was performed using Spearman’s rank correlation coefficient to analyze ranked data, and Mann-Whitney U to differentiate the means of different groups. A *p*-value < 0.05 was considered statistically significant. SPSS v. 10.0 software (SPSS, Chicago, IL, USA) was employed to analyze all data.

## Results

### The effects of SAHA on the phenotypes of ovarian carcinoma cells

**Figure 1:**
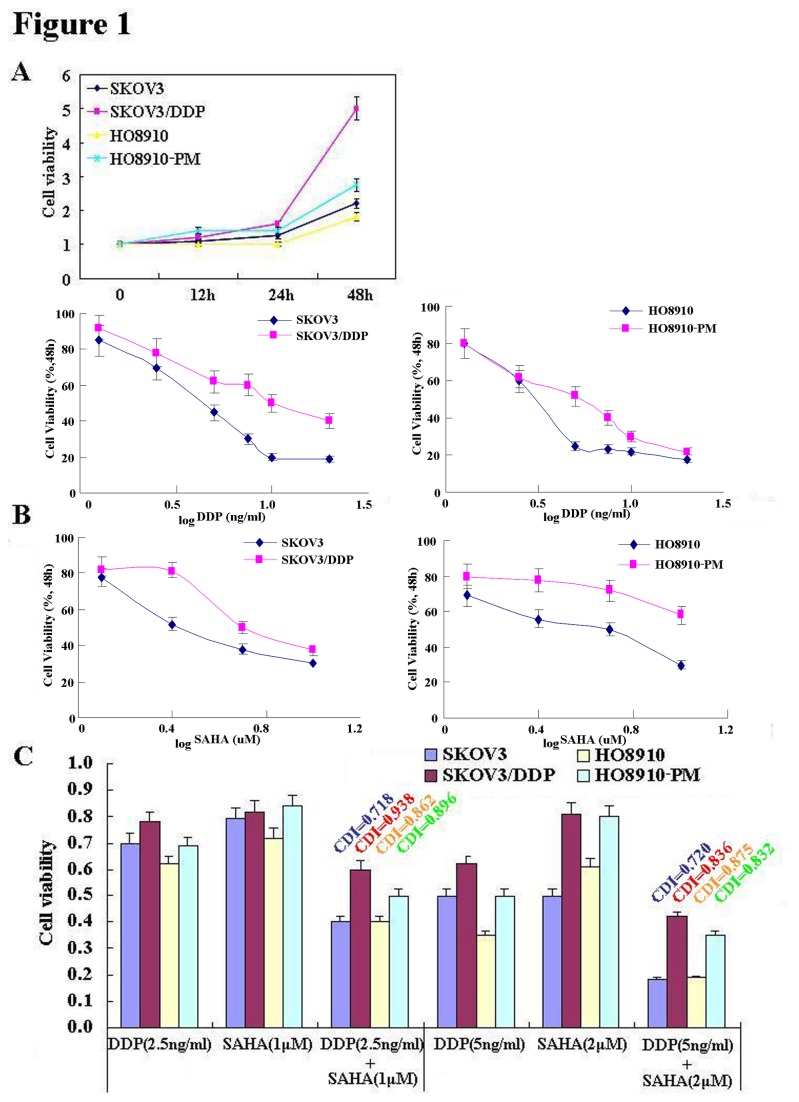
The effects of cisplatin (DDP) and SAHA on the proliferation of ovarian carcinoma cells. CCK-8 cell proliferation assays(n=3) showing that DDP (**A**) and SAHA (**B**) treatment of SKOV3 and SKOV3/DDP and HO8910 and HO8910-PM cell-line pairs induce cell death in a concentration- and time-dependent manner; furthermore, DDP and SAHA exhibit a synergistic effect when both treatments are applied in combination (**C**).

**Figure 2:**
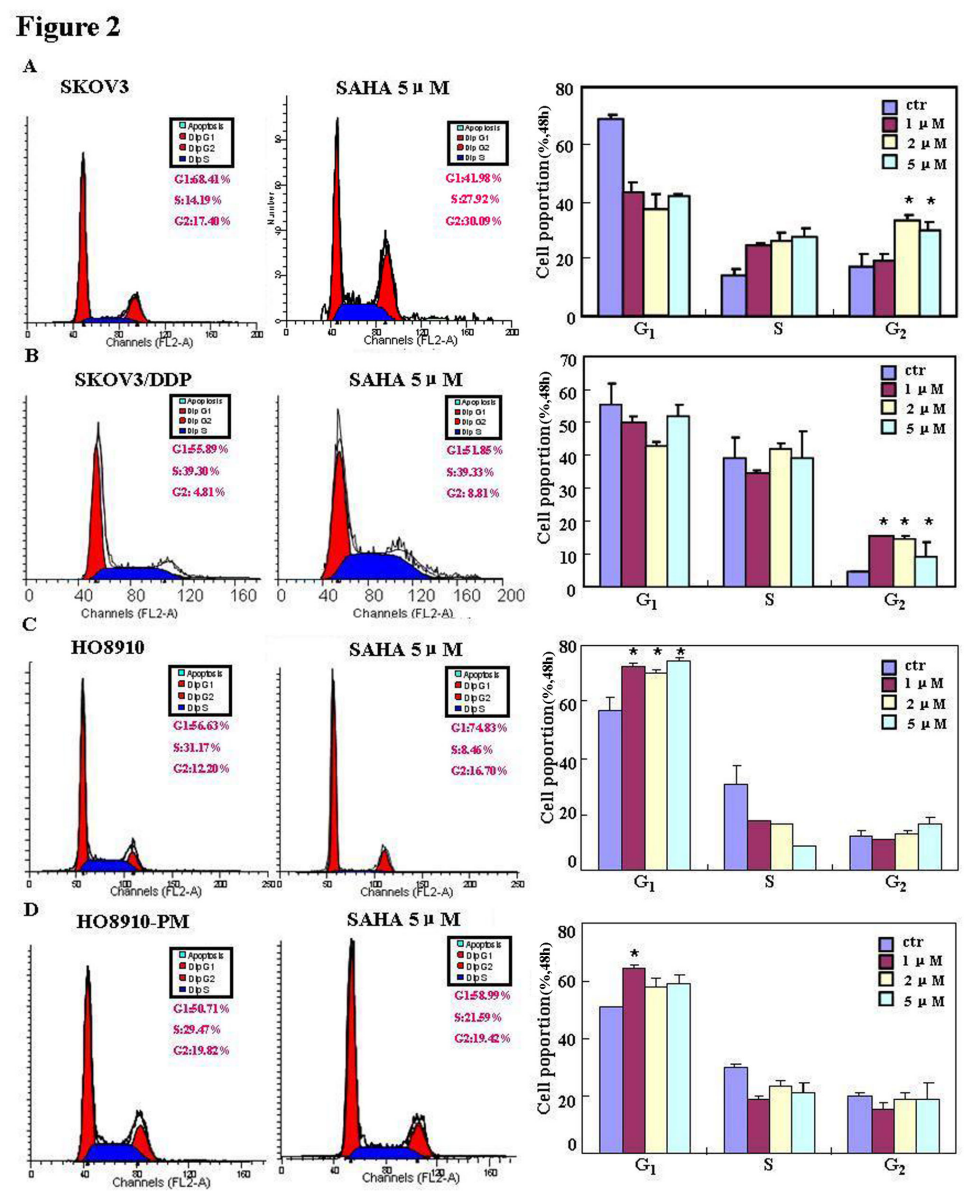
Effects of SAHA on the cell cycle of ovarian carcinoma cells. Flow cytometric analyses(n=3) of ovarian cell-line pairs after PI staining showing that SAHA treatment induces S/G_2_ arrest in SKOV3 (**A**) and SKOV3/DDP (**B**) cells; but conversely, induces G_1_ arrest in HO8910 (**C**) and HO8910-PM (**D**) cells.

**Figure 3:**
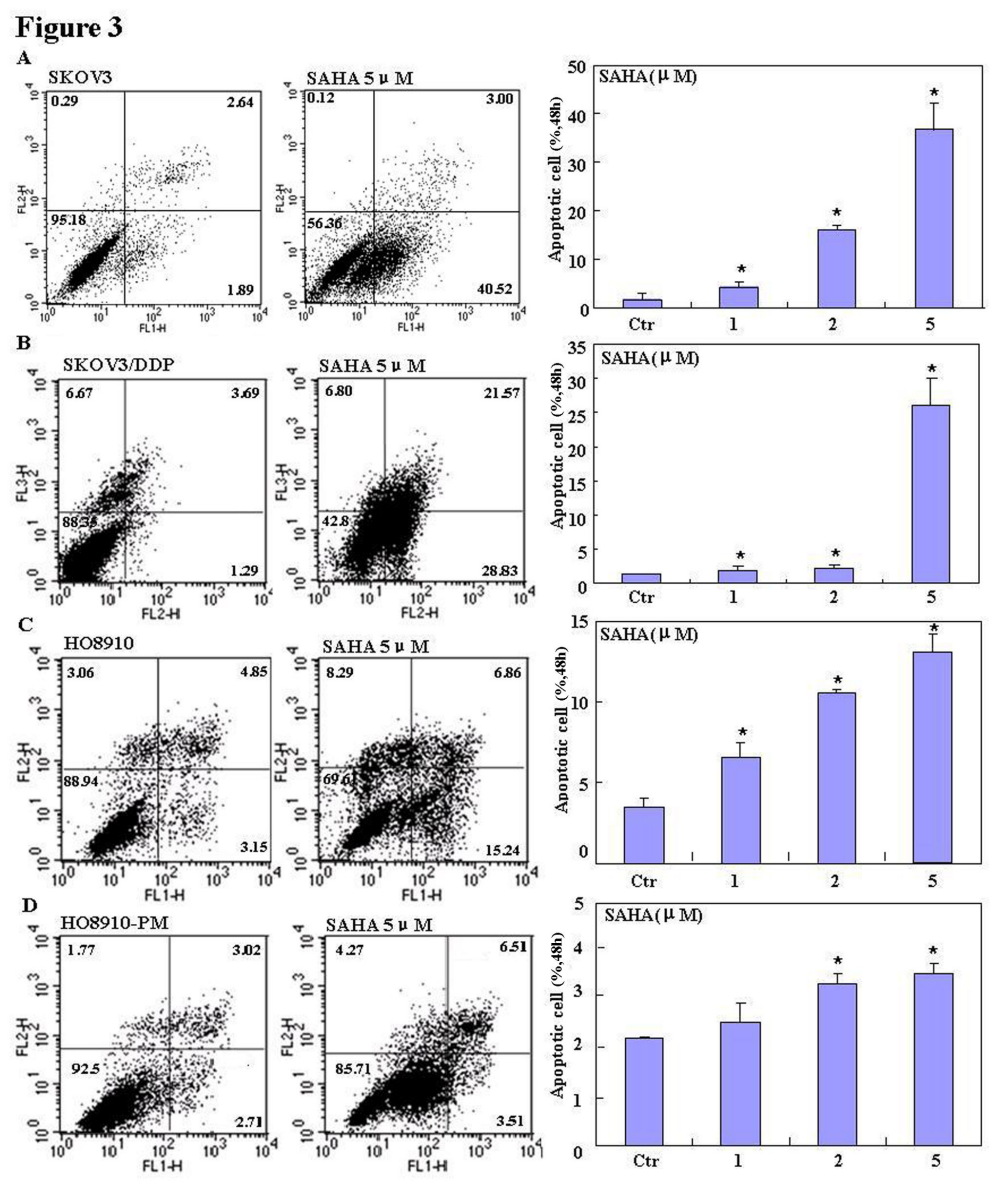
Effects of SAHA on apoptosis in ovarian carcinoma cells. Flow cytometric analyses(n=3) of ovarian cell-line pairs after Annexin IV staining showing that compared to negative controls, SAHA exposure results in higher levels of apoptosis in SKOV3 (**A**), SKOV3/DDP (**B**), HO8910 (**C**) and HO8910-PM (**D**) cells in a concentration-dependent manner.

**Figure 4:**
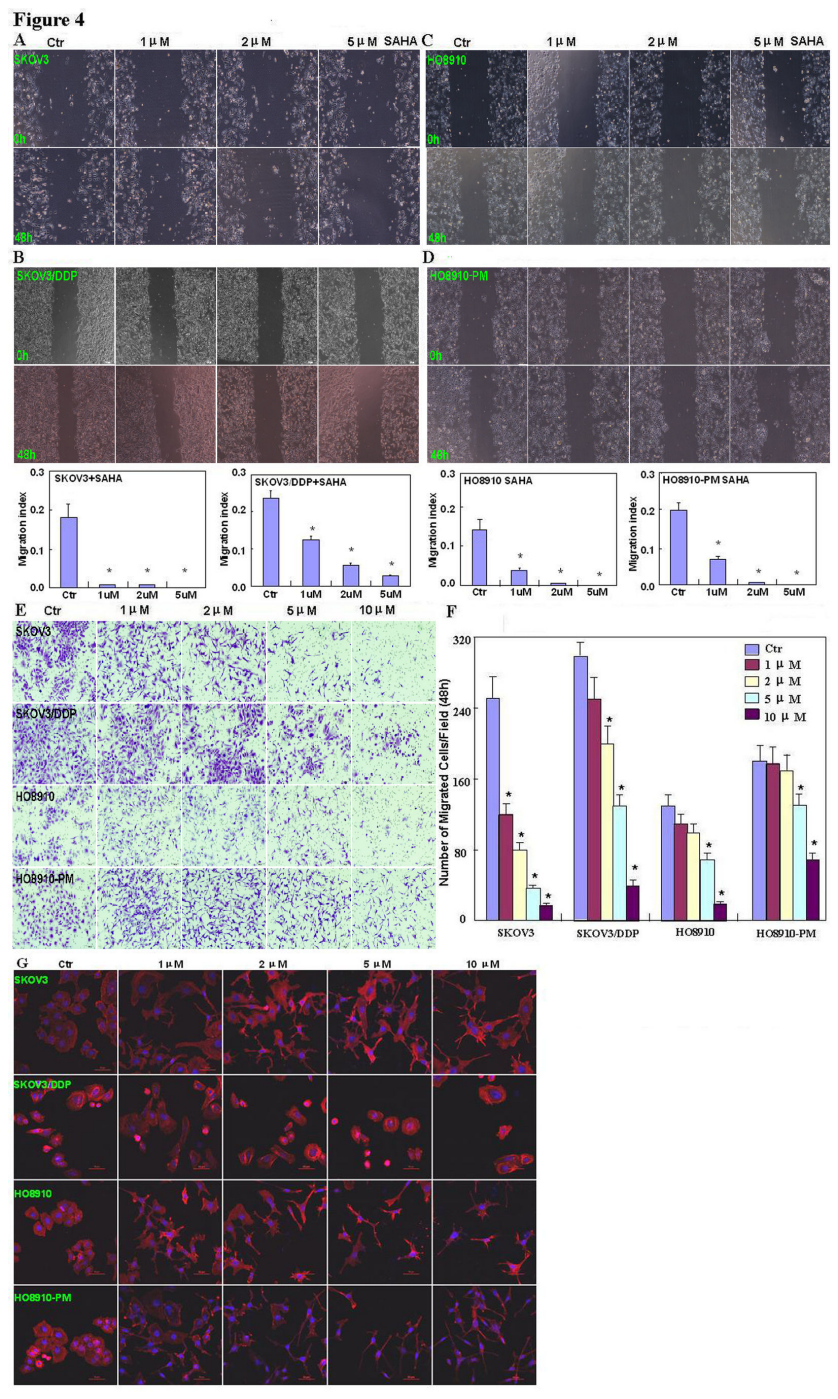
Effects of SAHA on the invasive ability of ovarian carcinoma cells. Wound healing assays showing that SAHA treatment decreases the ability of SKOV3 (**A**), SKOV3/DDP (**B**), HO8910 (**C**) and HO8910-PM (**D**) cells to migrate in a concentration-dependent manner(n=9); and reduces the invasive potential(E&F,n=3) and suppressed lamellipodia formation(**G**) of SKOV3, SKOV3/DDP, HO8910 and HO8910-PM cells.

 The pairs of cell lines (SKOV3 and SKOV3/DDP; HO8910 and HO8910-PM) were exposed to cisplatin (DDP) and subjected to CCK-8 proliferation assays. Although DDP induced cell death in all cells in a concentration- and time-dependent manner (Figure 1), SKOV3/DDP and HO8910-PM cells showed greater resistance to cisplatin than their parental cells(IC_50_ of SKOV3, SKOV3/DDP, HO8910, HO8910-PM was 5.2, 10.4, 3.98, 4.97 ng/ml). A similar effect was observed with SAHA treatment(IC_50_ of SKOV3, SKOV3/DDP, HO8910, HO8910-PM was 2.0, 4.9, 3.5, 11.2 μM); however, DDP and SAHA acted synergistically to suppress cell proliferation, since CDI value was 0.718, 0.938, 0.862, 0.896 (DDP: 2.5ng/ml;SAHA: 1μM) and 0.720, 0.836, 0.875, 0.832 (DDP: 5ng/ml;SAHA: 2μM) for SKOV3, SKOV3/DDP, HO8910 and HO8910-PM(Figure 1C). To further investigate the mechanism by which SAHA treatment may suppress proliferation in ovarian carcinoma cells, flow cytometric cell cycle analysis was performed. SAHA treatment induced G_2_ arrest in SKOV3 and SKOV3/DDP cells; conversely, SAHA induced G_1_ arrest in HO8910 and HO8910-PM cells (Figure 2). Flow cytometric apoptosis analysis showed that SAHA treatment induced apoptosis in SKOV3, SKOV3/DDP, HO8910 and HO8910-PM cells in a concentration-dependent manner (Figure 3); and the wound healing and matrigel transwell invasion assays showed that SAHA decreased cell migration and invasion in a concentration-dependent manner (Figure 4A–F). In addition, SAHA exposure suppressed lamellipodia formation in all four cell lines, as indicated by the loss of F-actin structure (Figure 4G).

### The mRNA and protein expression of phenotype-related molecules in ovarian carcinoma cells after exposure to SAHA

**Figure 5:**
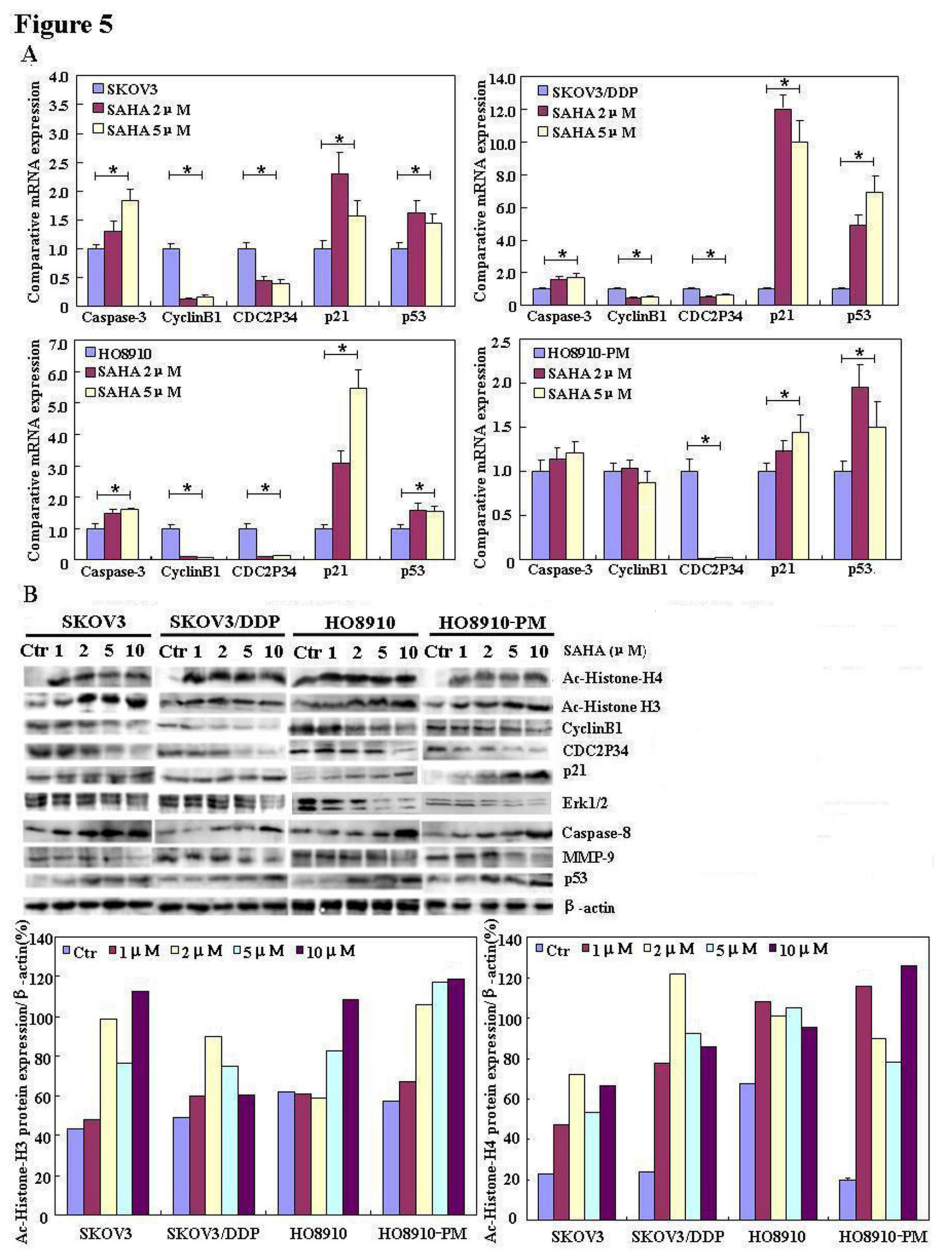
The mRNA and protein expression profiles of ovarian carcinoma cells treated with SAHA. Effects of SAHA exposure on the mRNA expression levels of phenotype-related molecules, and their corresponding proteins, in the ovarian carcinoma cell-line pairs: (A,n=3) real-time RT-PCR and (**B**) Western blot analysis. * *P* < 0.05.

After treatment with SAHA, the mRNA expression levels of *Cyclin B1* and *CDC2P34* in SKOV3, SKOV3/DDP, HO8910 and HO8910-PM cells were lower than those observed in control cells. In contrast, the mRNA expression levels of Caspase-3, *p21* and *p53* were higher than those of control cells (Figure 5A). Western blot analysis of protein expression levels (Figure 5B) showed that SAHA up-regulated acetyl-histones H3 and H4, Caspase-8, and p53 protein expression, and down-regulated CDC2P34, Erk1/2, CyclinB1 and MMP-9 (matrix metalloproteinase-9) protein expression, in both cell-line pairs. Although p21 was up-regulated by SAHA in both HO8910 and HO8910-PM cells, there was no change in p21 expression in SKOV3 or SKOV3/DDP cells following SAHA treatment.

### The association of acetylation of histone H3 and H4 with the pathogenesis and clinicopathological parameters of ovarian carcinoma

**Figure 6:**
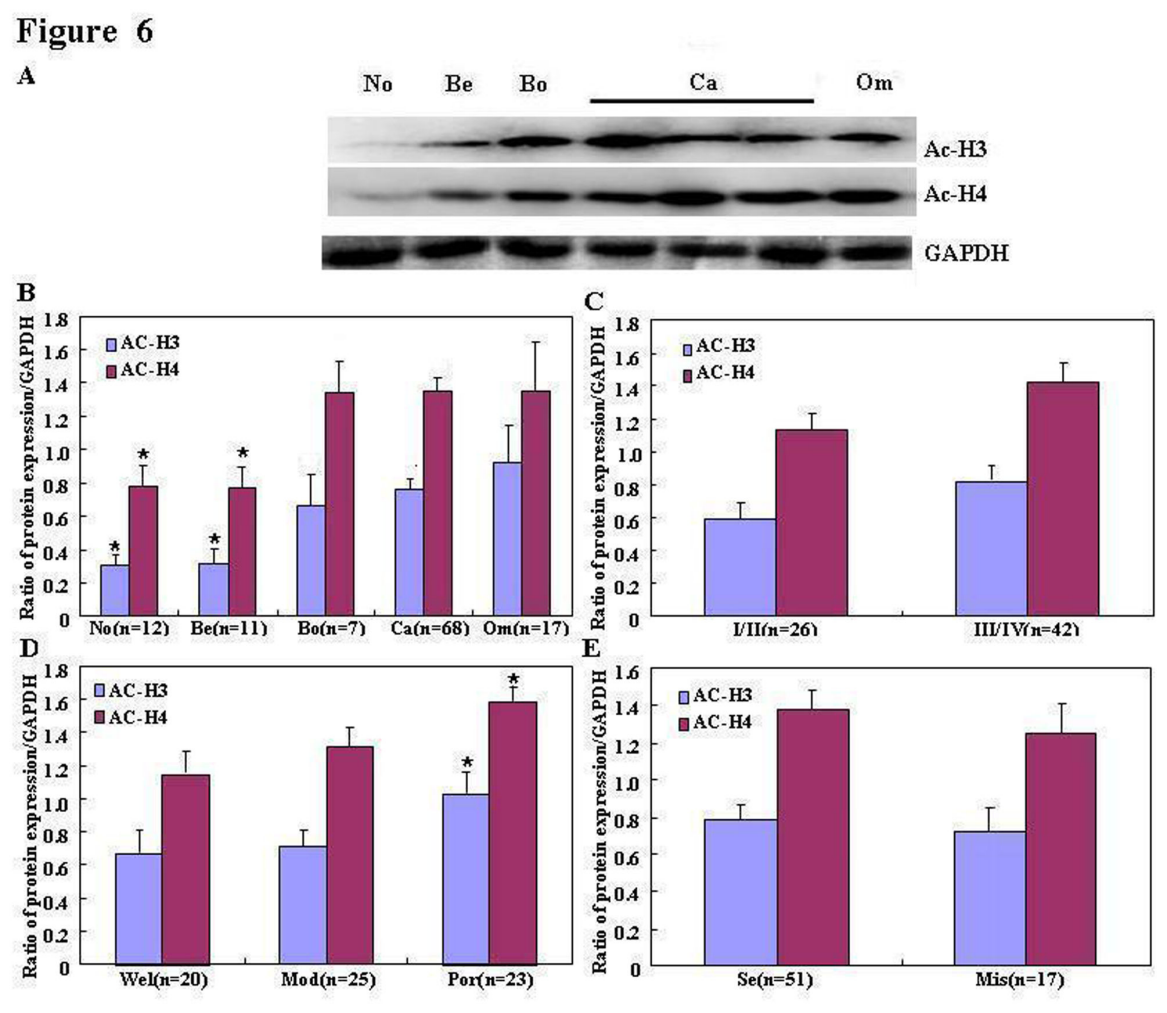
Correlation between acetylation of histone H3 and H4 and the pathogenesis and aggressiveness of ovarian carcinoma cells. Acetyl-histone H3 and H4 proteins were analyzed by (**A**) Western blotting in normal ovarian tissues (No; *n* = 12), benign tumors (Be; *n* = 11), borderline tumors (Bo; *n* = 7), primary carcinomas (Ca; *n* = 68) and metastatic carcinomas in the omentum (n = 17). The expression levels of both acetyl-histone H3 and H4 are significantly higher in ovarian carcinoma than in benign tumors and normal ovarian tissues, and in borderline tumor than in normal ovarian tissues (B; *P* < 0.05); and are positively linked to differentiation (**D**), but not to FIGO staging (**C**) or pathological subtyping in ovarian carcinoma (**E**).

Acetylation of histone H3 and H4 were detected by Western blotting in normal ovarian tissue, benign and borderline tumors, and primary carcinoma samples (Figure 6A). Densitometric analyses showed that the expression of both acetyl-histone H3 and H4 were significantly higher in primary carcinoma than benign tumor cells and normal ovarian tissues, and in borderline tumor than in normal ovarian tissues (*P* < 0.05; Figure 6B); and in poorly-differentiated adenocarcinoma than in well- and moderately- differentiated adenocarcinoma (*P* < 0.05; Figure 6D). Although expression of acetylation of histone H3 and H4 were positively correlated in ovarian carcinoma (*P* < 0.05; Figure 6F), no correlations were observed between acetylation of histone H3 and H4 and FIGO staging or pathological subtyping in ovarian carcinoma (*P* > 0.05; Figure 6C and E, respectively).

**Figure 7:**
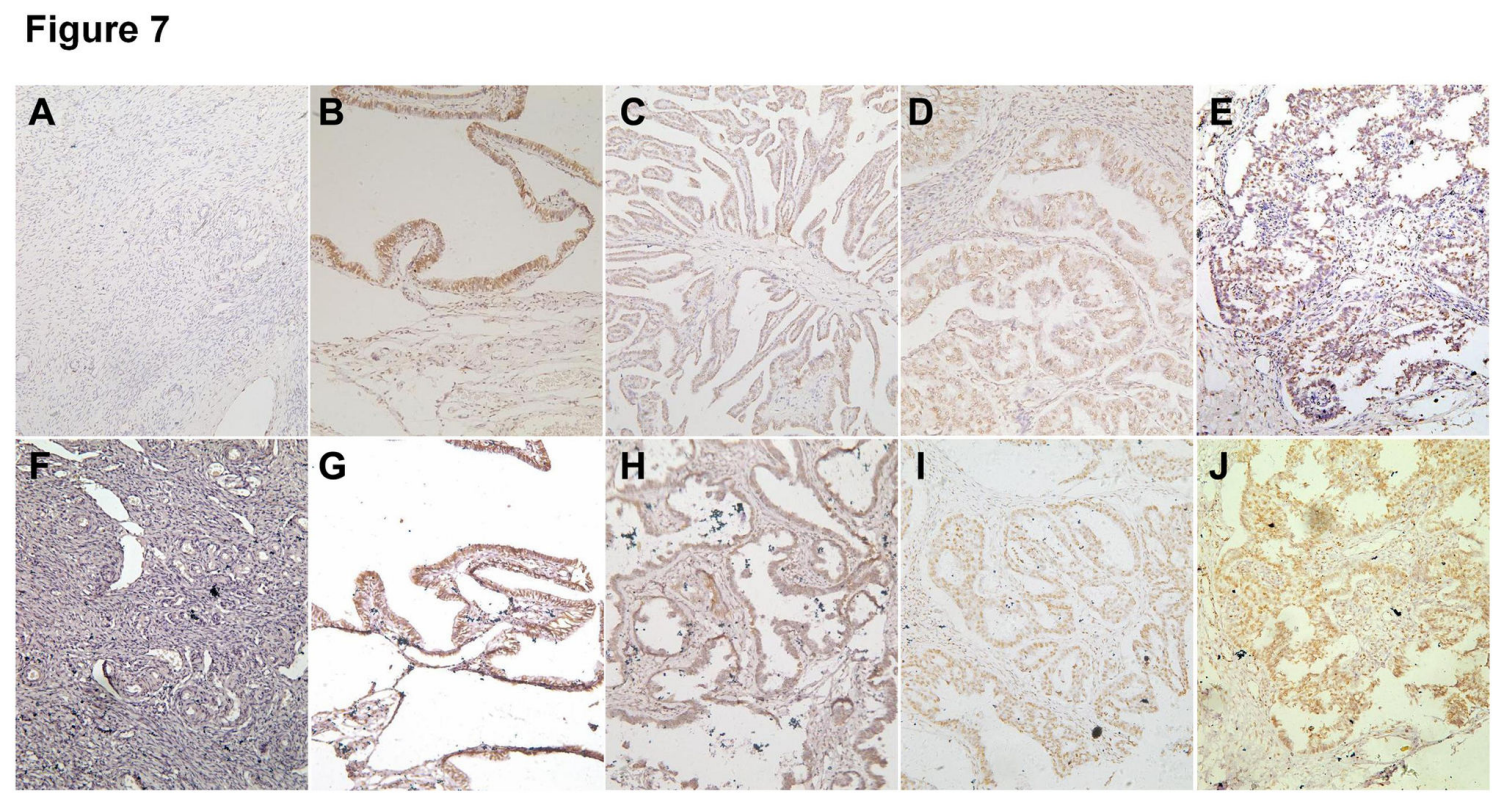
Immunohistochemical analysis of acetyl-histone H3 and H4 expression levels in ovarian lesions. Acetyl-histone H3 and H4 proteins are absent in the majority of the normal ovarian samples (**A**, **F**). In contrast, they are positively expressed in the nuclei of benign ovarian tumor cells (**B**, **G**), borderline tumor cells (**C**, **H**), and serous (**D**, **I**) and mucinous (**E**, **J**) adenocarcinoma.

Immunohistochemistry showed that acetyl-histones H3 and H4 were distributed in the nuclei of normal ovarian tissues, benign and borderline tumor cells and primary carcinomas (Figure 7). Based on expression frequency and density measurements, acetylation of histone H3 and H4 were found to be more highly expressed in primary ovarian carcinoma than normal ovarian tissues or benign tumor cells (*P* < 0.05; Table 1); and in borderline tumor cells than in normal ovarian tissue (*P* < 0.05; Table 1). In addition, immunohistochemistry showed that acetylation of histone H3 and H4 were positively linked to differentiation and expression of Ki-67, a proliferation marker, but not linked to patient age, histological classification or FIGO staging (*P* < 0.05; Table 2). 

**Table 1:** Changes in acetyl-histone H3 and H4 expression levels during ovarian epithelial carcinogenesis.

**Group**	**n**	**Ac-H3 expression**					**Ac-H4 expression**				
		**-**	**+**	**++**	**+++**	**%**	**-**	**+**	**++**	**+++**	**%**
Normal ovarian tissue	23	13	1	3	6	43.5	17	0	1	5	26.1
Benign ovarian tumor	18	9	2	3	4	50.0	11	3	2	2	38.9
Borderline ovarian tumor	10	3	1	3	4	72.7*	1	3	0	6	90.9*
Primary ovarian carcinoma	174	36	26	43	69	79.3*	34	13	23	104	80.5*
Metastatic carcinoma in omentum	35	10	3	4	18	71.4	7	2	11	15	80.0

* *P* < 0.05 compared to normal ovarian and benign tumor samples.

**Table 2:** Relationship between acetyl-histone H3 and H4 expression levels and the clinicopathological features of ovarian carcinoma.

**Clinicopathological feature**	**n**	**Ac-H3 expression**						**Ac-H4 expression**					
		**-**	**+**	**++**	**+++**	**%**	**p**	**-**	**+**	**++**	**+++**	**%**	**p**
**Age (years)**							0.787						0.796
<56	75	18	11	15	31	76.0		16	6	6	47	78.7	
≥56	99	18	15	28	38	81.8		18	7	17	57	81.8	
**Pathological classification**							0.407						0.243
Serous adenocarcinoma	125	25	23	30	47	80.0		25	12	17	71	80.0	
Mucinous adenocarcinoma	15	5	1	3	6	66.7		6	0	1	8	60.0	
Miscellaneous subtypes	34	6	2	10	16	82.4		3	1	5	25	91.2	
**FIGO staging**							0.766						0.076
I–II	64	15	5	21	23	76.6		10	3	7	44	84.4	
III–IV	110	21	21	23	46	80.9		24	10	16	60	78.2	
**Differentiation**							0.009						0.048
Well-differentiated	40	11	4	8	17	72.5		8	4	5	23	80.0	
Moderately-differentiated	61	17	13	14	17	72.1		16	5	10	30	73.8	
Poorly-differentiated	73	8	9	21	35	89.0		10	4	8	51	86.3	
**Ki-67 expression**							0.004						0.024
-	64	24	10	11	19	62.5		21	6	5	32	67.2	
+	37	6	3	13	15	83.8		7	1	6	23	81.1	
++	35	4	6	9	16	88.6		4	4	6	21	88.6	
+++	38	2	7	10	19	94.7		2	2	6	28	94.7	

% = positive rate

## Discussion

SAHA anti-tumor activity has been reported in cells from a number of hematogenous and solid malignancies *in vitro*, including leukemia, mantle cell lymphoma, hepatoma, pancreatic carcinoma, breast cancer, prostate cancer, colon cancer and chondrosarcoma [7-16]. In a clinical phase I study by Kelly et al. [17], daily administration of intravenous SAHA was well tolerated; dose limiting toxicities were mainly hematologic, with only 4% of patients with solid tumors experiencing Grade 3 thrombocytopenia, and only 8% showing Grade 3 anemia. When SAHA was clinically employed to treat hematologic malignancies, such as advanced or recurrent cutaneous T-cell lymphoma, it was found to be effective, with good oral bioavailability and an acceptable safety profile [18]. However, in a phase II trial, recurrent ovarian cancer did not show significant susceptibility to SAHA when it was used as a single agent [19].

Evidence from experimental studies has shown that SAHA reduces cell viability in ovarian cancer cells in both a dose- and time-dependent manner [20]. The aim of the present study was to elucidate the effect and related molecular mechanisms of SAHA on the aggressive phenotypes of ovarian carcinoma cells. Previous studies have reported that a combination of 5-aza-20-deoxycytidine (5-azadC) or paclitaxel with SAHA inhibited ovarian cancer growth while inducing apoptosis, G_2_/M phase arrest and autophagy [21,22]. We selected two pairs of ovarian carcinoma cell lines, based on their drug resistance and invasive abilities (SKOV3 and SKOV3/DDP; and HO8910 and HO8910-PM, respectively). Both the drug-resistant (SKOV3/DDP) and highly-invasive (HO8910-PM) cells showed increased cisplatin resistance, which may be due to their greater proportion of stem cells (tumor initiating cells) or more stem cell-like properties of these cells (IC_50_ of SKOV3, SKOV3/DDP, HO8910, HO8910-PM was 5.2, 10.4, 3.98, 4.979 ng/ml). In agreement with previous reports on the apoptotic effects of SAHA in P-glycoprotein-positive and -negative cells (13), SAHA induced cell death in both these cell-line pairs, although SKOV3/DDP and HO8910-PM cells showed greater resistance to SAHA compared to their parental cells(IC_50_ of SKOV3, SKOV3/DDP, HO8910, HO8910-PM was 2.0, 4.9, 3.5, 11.2 μM). Although SAHA induced apoptosis and inhibited migration (Cells were photographed at 0,12,24,48,72 h, however, we found that the wound healing rate was significantly changed at 48h), invasion and the ability to form lamellipodia in ovarian carcinoma cells when used alone; a combination of cisplatin and SAHA exhibited synergistic cytotoxic effects, suggesting that this dual effect may more effective in the treatment of ovarian cancer than SAHA alone. Taken together, these findings suggest that SAHA may inhibit the aggressive phenotypes of ovarian carcinoma cells, including cisplatin-resistant or highly- invasive cells.

Here, we found that SKOV3 cells and their cisplatin-resistant variant, SKOV3/DDP, showed no change in expression of p21 upon SAHA treatment; whereas p21 was upregulated in HO8910 cells and their highly-invasive variant, HO8910-PM, after exposure to SAHA. In addition, SAHA induced G_2_/M arrest in SKOV3 and SKOV3/DDP cells, but conversely, induced G_1_ arrest in HO8910 and HO8910-PM cells. It has been suggested that G_1_ phase arrest may result from overexpression of p21cip1/waf1 due interactions with various cyclins and cyclin-dependent kinases (CDK) [23]. This may offer an explanation for the cell specific effects of SAHA in relation to the cell cycle. Changes in cyclin B1 and Cdc2 (p34) expression may contribute to this specificity as both are involved in mitotic events: Cyclin B1 is involved in the early events of mitosis by interacting with Cdk1; and Cdc2 (p34) is an M-phase promoting factor that induces entry into mitosis [24,25]. In addition, caspase-3 is implicated in the initiation of apoptosis when activated by caspases-8, -9 and -10 via extrinsic and intrinsic pathways [26]. As such, increasing the expression of these factors via SAHA exposure may contribute to the induction of apoptosis in ovarian carcinoma cells. Our results also showed that SAHA decreased expression of Erk1/2 and MMP-9, and increased expression of p53, which in turn altered the proliferation and apoptosis in ovarian carcinoma cells. In response to mitogens, growth factors and cytokines, activated Erk1/2 is involved in cell survival by downregulating pro-apoptotic molecules [27]; p53 is a master switch that coordinates stress signals associated with apoptosis and cell cycle arrest [26]; and MMP-9 plays a role in cancer metastasis via breakdown of the extracellular matrix [28].

Epigenetic modifications, such as methylation, acetylation and chromatin remodeling, regulate gene expression; as such, epigenetic events play central roles in carcinogenesis and tumor development. Here, we found that SAHA promoted acetylation of histones H3 and H4 during SAHA-induced apoptosis in ovarian carcinoma cells. To investigate the clinicopathological significance of both of these acetyl-histone proteins, we carried out immunohistochemistry and Western blot analysis. These showed, for the first time, that expression of both acetylation of histone H3 and H4 were significantly higher in ovarian carcinoma than in benign tumor and normal ovarian tissues, and in borderline tumor than in normal ovarian tissues. These findings suggest that the overexpression of these marker proteins may be a reactive response which plays an important role in ovarian carcinogenesis. Both proteins were positively linked to the differentiation and Ki-67 expression of ovarian carcinoma cells, indicating that they might be closely linked to differentiation and proliferation in ovarian carcinoma. Consistent with these observations, immunostaining and Western blot analysis showed a positive correlation between acetyl-histones H3 and H4. Minardi et al. [29] reported that the mean percentage of anti-acetyl-histone H3 (Lys9) was significantly lower in renal cell carcinoma (RCC) tumor tissue than the adjacent normal tissues, and also lower in grade 3 and 4 than grade 1 and 2 pT1a RCCs, suggesting that histone acetylation is an early event in RCC and correlates with tumor aggressiveness.

In conclusion, we have shown that SAHA potentiates cisplatin-induced cytotoxicity in ovarian cancer cell lines, including cisplatin-resistant and highly-invasive cells, by inducing apoptosis and suppressing proliferation, migration, invasion and lamellipodia formation. Our findings suggest that the mechanism of SAHA may involve modulation of the acetylation of histone H3 and H4, and/or phenotype-related molecules. Aberrant acetylation of these histones may be involved in the tumorigenesis and differentiation of ovarian carcinoma; therefore, SAHA could potentially be employed as a chemotherapeutic agent in ovarian carcinoma clinical practice. However, its anti-tumor mechanisms need to be clarified further, and a clinical trial performed in the future.

## Supporting Information

File S1 **Table S1, Primer sequences selected for real-time RT-PCR. Table S2, Antibodies used for Western blotting.**
(DOC)
