# Supplementary material for: The Anti-Tumor Effects and Molecular Mechanisms of Suberoylanilide Hydroxamic Acid (SAHA) on the Aggressive Phenotypes of Ovarian Carcinoma Cells
Source: PLoS One. 2013 Nov 13;8(11):e79781. doi: 10.1371/journal.pone.0079781 (PMC3827455; doi:10.1371/journal.pone.0079781)
Supplement: File S1 — Table S1, Primer sequences selected for real-time RT-PCR. Table S2, Antibodies used for Western blotting. (DOC) [file pone.0079781.s001.doc]

**Table S1.** Primer sequences selected for real-time RT-PCR

| **Name** | **Primer sequence** | **Distribution** | **AT (^o^C)** | **Product size (bp)** | **Extension time (s)** |
| --- | --- | --- | --- | --- | --- |
| *Caspase-3* | F: 5'-TGGCATTGAGACAGACA-3'  R: 5'-GGCACAAAGCGACTG-3' | NM_004346 773-926 | 60 | 154 | 34 |
| *Cyclin B1* | F: 5'-GCACTTTCCTCCTTCT-3'  R: 5'-CGATGTGGCATACTTG-3' | NM_031966 1264-1474 | 60 | 211 | 34 |
| *CDC2p34* | F: 5'-TCGCACTTGGCTTCA-3'  R: 5'-TTAGTCAATGGGTATGGTA-3' | NM_001786 23-140 | 60 | 118 | 34 |
| *p21* | F: 5'-ACTGTCTTGTACCCTTGTGCC-3'  R: 5'-AAATCTGTCATGCTGGTCTGC-3' | [NM_000389.3](http://www.ncbi.nlm.nih.gov/entrez/viewer.fcgi?db=nucleotide&id=169790847) 464-571 | 60 | 108 | 34 |
| *p53* | F: 5'-ACCCAGGTCCAGATGAAG-3'  R: 5'-GCAAGAAGCCCAGACG-3' | NM_001126118.1 489-662 | 60 | 174 | 34 |
| *GAPDH* | F: 5'-CAATGACCCCTTCATTGACC-3'  R: 5'-TGGAAGATGGTGATGGGATT-3' | NM_002046.3 201-335 | 60 | 135 | 34 |

AT = annealing temperature

**Table S2.** Antibodies used for Western blotting

| **Name** | **Source** | **Company** |
| --- | --- | --- |
| Ac-Histone H4 (Lys 8) | Rabbit | Santa Cruz Biotechnology |
| Ac-Histone H3 (Lys 9/14) | Rabbit | Santa Cruz Biotechnology |
| Cyclin B1 (GNS1) | Mouse | Santa Cruz Biotechnology |
| Phospho-Cdc2 p34 (Tyr 15) | Goat | Santa Cruz Biotechnology |
| p21 (F-5) | Mouse | Santa Cruz Biotechnology |
| Erk1/2 (MK1) | Mouse | Santa Cruz Biotechnology |
| Caspase-3 p20 (N-19) | Goat | Santa Cruz Biotechnology |
| MMP-9 (H-129) | Rabbit | Santa Cruz Biotechnology |
| p53 | Rabbit | Santa Cruz Biotechnology |
| β-actin (C-4) | Mouse | Santa Cruz Biotechnology |
